# Supplementary material for: Remdesivir Treatment for COVID 19 in Pregnant Patients with Moderate to Severe Symptoms: Serial Case Report
Source: Infect Dis Rep. 2021 May 17;13(2):437–43. doi: 10.3390/idr13020042 (PMC8162322; doi:10.3390/idr13020042)
Supplement: Supplementary file 1 [file idr-13-00042-s001.zip › idr-1163839-supplementary.pdf]

| Laboratory Component | | A | | | | | | B | | C | D | | | E | |
|---|---|---|---|---|---|---|---|---|---|---|---|---|---|---|---|
| Reference Range and Units | Reference Range and Units | 20/12/20 | 24/12/20 | 21/2/21 | D1 | D2 | D3 | D1 | D2 | D1 | D1 | D2 | D3 | 11/1/21 | D |
| Oxygen Requirement (NC) | None | | | | | | | | | | | | | | |
| Hemoglobin (g/dL) | 11.7-15.7 | 12.9 | 11.3 | 12.7 | 12.1 | 11.4 | 12.7 | 13.6 | 12.7 | 12.0 | 10.3 | 10.7 | 10.1 | 10.5 | 10.1 |
| Erythrocytes ($10^6$/uL) | 3.80 – 4.80 | 4.33 | 3.72 | 3.97 | 4.14 | 3.87 | 4.34 | 4.25 | 3.97 | 4.63 | 3.95 | 4.08 | 3.81 | 3.65 | 3.53 |
| Thrombocytes ($10^3$/ uL) | 150-410 | 14.21 | 13.30 | 360 | 13.59 | 20.66 | 18.92 | 14.18 | 19.68 | 5.31 | 8.86 | 7.67 | 12.83 | 270 | 226 |
| WBC Count ($10^3$/uL) | 4.0-11.0 | 298 | 284 | 19.86 | 371 | 483 | 646 | 344 | 360 | 307 | 265 | 313 | 355 | 8.25 | 6.14 |
| MCV/VER (fL) | 83.0-101.0 | 88 | 86.3 | 89.9 | 85.3 | 84.2 | 86.9 | 88.7 | 89.9 | 79.3 | 79.5 | 79.4 | 79.5 | 84.1 | 83.0 |
| MCH/HER (pg) | 27.0-32.0 | 29.8 | 30.4 | 32.0 | 29.2 | 29.5 | 29.3 | 32.0 | 32.0 | 25.9 | 26.1 | 26.2 | 26.5 | 28.8 | 28.6 |
| MCHC/KHER (g/dL) | 31.5-34.5 | 33.9 | 35.2 | 35.6 | 34.3 | 35.0 | 33.7 | 36.1 | 35.6 | 32.7 | 32.8 | 33 | 33.3 | 34.2 | 34.5 |
| Basophil (%) | 0-2 | 0.5 | 0.5 | 0.3 | 0.6 | 0.5 | 0.1 | 0.8 | 0.3 | 0.4 | 0.2 | | | 0.4 | 0.2 |
| Eosinophil (%) | 1-6 | 0.1 | 0.2 | 0.0 | 0.0 | 0.0 | 0.1 | 0.0 | 0.0 | 1.5 | 0.1 | | | 0.0 | 0.2 |
| Neutrophil (%) | 40.0-80.0 | 78.1 | 87.3 | 84.9 | 89.0 | 85.3 | 91.2 | 78.1 | 84.9 | 69.4 | 72.7 | | | 73.3 | 70.8 |
| Lymphocyte (%) | 20-40 | 14.2 | 8.3 | 10.6 | 8.2 | 9.7 | 6.3 | 15.2 | 10.6 | 20.0 | 13.2 | | | 13.7 | 21.3 |
| Monocyte (%) | 2-10 | 0.0 | 3.7 | 4.2 | 2.2 | 4.5 | 2.3 | 5.9 | 4.2 | 8.7 | 13.8 | | | 12.6 | 7.5 |
| Neutrophiil Count ($10^3$/uL) | 1.70-7.50 | 11.10 | 11.63 | 16.73 | 12.10 | 17.61 | 17.26 | 11.09 | 16.73 | 3.69 | 6.44 | | | 6.05 | 4.35 |
| Lymphocyte Count ($10^3$/uL) | 1.00-3.20 | 2.02 | 1.10 | 2.08 | 1.11 | 2.01 | 1.20 | 2.15 | 2.08 | 1.06 | 1.17 | | | 1.13 | 1.31 |
| NLCR | None | 5.50 | 10.57 | 8.04 | 10.90 | 8.76 | 14.28 | 5.16 | 8.04 | 3.48 | 5.50 | | | 5.35 | 3.32 |
| CRP (mg/dL) | <0.50 | 58.1 | | | 226.6 | | | 41.6 | | 10.7 | 12.3 | 31.9 | 8.62 (Hs) | 17.9 | |
| Albumin (g/dL) | 3.50-5.20 | 3.71 | | | | | | | | | | | | | |
| Creatinine (mg/dL) | 0.55 – 1.02 | 0.50 | 0.50 | | | | 0.50 | 0.80 | | 0.90 | 0.60 | 0.50 | | | |
| eGFR (mL/min/1.73m²) | 86.0- 128.0 | 130.3 | 130.3 | | | | 130.3 | 100.7 | | 85.5 | 123.6 | 131.2 | | | |
| AST (U/L) | 10-35 | 23 | 53 | | | | 21 | 306 | | 30 | 25 | 21 | | | |
| ALT (U/L) | 0-55 | 23 | 55 | | | | 57 | 273 | | 25 | 24 | 24 | | | |
| Prothrombin Time (seconds) | 9.8-12.6 | 9.7 | | | | | | 10.9 | | 9.2 | 10.1 | | | | |
| Activated Partial Thromboplastin Time (seconds) | 31.0 - 47.0 | 33.4 | | | 35.3 | | | 31.6 | | 36.0 | 37.0 | | 32.9 | | 37.9 |
| D-dimer (ug/mL) | <440 | | 1110 | | 930 | | 3510 | 930 | | 2600 | 1500 | | 3120 | 2020 | 1250 |
| Fibrinogen (mg/dL) | 200-400 | | | | | | | | | 579.7 | 502.3 | | | | |
| Ferritin (ng/mL) | 20.0 – 200.0 | | | | | | 158.84 | | | | | | | | |
| LDH (U/L) | 135-214 | | 240 | | | | 287 | | | | | | | | |
| Procalcitonin (ng/mL) | <0.05 | 0.06 | | | | | | 0.53 | | 0.08 | 0.06 | | | | |
| Na (mEq/L) | 136-145 | 136 | | | | 141 | | 136 | | 137 | 129 | 131 | | | |
| K (mEq/L) | 3.5-5.1 | 3.8 | | | | 3.2 | | 2.9 | | 4.6 | 4.0 | 3.9 | | | |
| Cl (mEq/L) | 98-107 | 103.1 | | | | 102.0 | | 95.0 | | 104.6 | 98.5 | 100 | | | |

| | | | | | | | | | | | | | | | |
|---|---|---|---|---|---|---|---|---|---|---|---|---|---|---|---|
| **Blood Glucose (mg/dL)** | 60-14 | 111 | 109 | | | | | 99 | | 72 | 82 | | | | |
| **Ureum (mg/dL)** | 15-40 | 14.7 | | | | | 35.4 | 19.2 | | 23 | 16.0 | 16.0 | | | |
| **Albumin (g/dL)** | 3.50-5.20 | | | | | | | | | | | 3.33 | | | |
| **Uric Acid (mg/dL)** | 2.6-6 | | 2.9 | | | | | | | | | | | | |
| **SARS COV-2 Real Time PCR** | Negative | | Positive | | | | | | | | | | Positive | Positive | Positive |
| **HbsAg** | Non-Reative | | | | | | | | | Non Reactive | | Non reactive | | | |
